# Supplementary material for: Proteomic Analyses Reveal New Insights on the Antimicrobial Mechanisms of Chitosan Biopolymers and Their Nanosized Particles against Escherichia coli
Source: Int J Mol Sci. 2019 Dec 28;21(1):225. doi: 10.3390/ijms21010225 (PMC6981525; doi:10.3390/ijms21010225)
Supplement: Supplementary file 1 [file ijms-21-00225-s001.zip › Supplementary material Table S4.docx]

**Supplementary material 4:**

**SWATH acquisition:**

The information dependent acquisition (IDA) experiments were performed for each pooled samples in a total of two acquisitions per pool. The mass spectrometer was set to scanning full spectra (350-1250 m/z) for 250ms, followed by up to 100 MS/MS scans (100–1500 m/z from a dynamic accumulation time – minimum 30 ms for precursor above the intensity threshold of 1000 – in order to maintain a cycle time of 3.298 s). Candidate ions with a charge state between +2 and +5 and counts above a minimum threshold of 10 counts per second were isolated for fragmentation and one MS/MS spectra was collected before adding those ions to the exclusion list for 15 seconds (mass spectrometer operated by Analyst® TF 1.7, ABSciex®). Rolling collision was used with a collision energy spread of 5.

For SWATH-MS based experiments, the mass spectrometer was operated in a looped product ion mode [[1](#_ENREF_1)] and the same chromatographic conditions used as in the IDA run described above. A set of 168 windows (Table S1) of variable (width containing 1 m/z for the window overlap) was constructed covering the precursor mass range of 350-1250 m/z. A 50 ms survey scan (350-1500 m/z) was acquired at the beginning of each cycle for instrument calibration and SWATH MS/MS spectra were collected from 100–1500 m/z for 19 ms resulting in a cycle time of 3.2911 s from the precursors ranging from 350 to 1250 m/z. The collision energy for each window was determined according to the calculation for a charge +2 ion centered upon the window with variable collision energy spread (CES) according with the window.

A specific library of precursor masses and fragment ions was created by combining all files from the IDA experiments, and used for subsequent SWATH processing. Libraries were obtained using ProteinPilot^TM^ software (v5.1, ABSciex®), using the following parameters: i) search against a database composed the UniProt reviewed database of *Escherichia Coli* (downloaded at May 2019), and MBP-GFP (IS); ii) iodoacetamide alkylated cysteines as fixed modification; iii) trypsin as digestion type. An independent False Discovery Rate (FDR) analysis using the target-decoy approach provided with Protein Pilot software was used to assess the quality of the identifications and positive identifications were considered when identified proteins and peptides reached a 5% local FDR [[2](#_ENREF_2),[3](#_ENREF_3)].

Data processing was performed using SWATH^TM^ processing plug-in for PeakView^TM^ (v2.0.01, ABSciex®) [[4](#_ENREF_4)]. After retention time adjustment using the MBP-GFP peptides, up to 15 peptides, with up to 5 fragments each, were chosen per protein, and quantitation was attempted for all proteins in library file that were identified from ProteinPilot™ searches.

Peptides’ confidence threshold was determined based on a FDR analysis using the target-decoy approach and those that met the 1% FDR threshold in at least three of the four biological replicates, and with at least 3 transitions were retained. Peak areas of the target fragment ions (transitions) of the retained peptides were extracted across the experiments using an extracted-ion chromatogram (XIC) window of 5 minutes with 100 ppm XIC width.

The levels of the proteins were estimated by summing all the transitions from all the peptides for a given protein that met the criteria described above (an adaptation of [[5](#_ENREF_5)]) and normalized to the total intensity of each sample.

**Table S1 – SWATH-MS method.**

|  | m/z range | Width (Da) | CES |
| --- | --- | --- | --- |
| **Window 1** | 349.5-394.1 | 44.6 | 5 |
| **Window 2** | 393.1-415.3 | 22.2 | 5 |
| **Window 3** | 414.3-427 | 12.7 | 5 |
| **Window 4** | 426-431.9 | 5.9 | 5 |
| **Window 5** | 430.9-436 | 5.1 | 5 |
| **Window 6** | 435-439.6 | 4.6 | 5 |
| **Window 7** | 438.6-443.2 | 4.6 | 5 |
| **Window 8** | 442.2-446.3 | 4.1 | 5 |
| **Window 9** | 445.3-449.9 | 4.6 | 5 |
| **Window 10** | 448.9-453.1 | 4.2 | 5 |
| **Window 11** | 452.1-456.2 | 4.1 | 5 |
| **Window 12** | 455.2-459.4 | 4.2 | 5 |
| **Window 13** | 458.4-462.4 | 4 | 5 |
| **Window 14** | 461.1-465.2 | 4.1 | 5 |
| **Window 15** | 464.2-468.4 | 4.2 | 5 |
| **Window 16** | 467.4-471.4 | 4 | 5 |
| **Window 17** | 470.1-474.2 | 4.1 | 5 |
| **Window 18** | 473.2-477.2 | 4 | 5 |
| **Window 19** | 475.9-480.1 | 4.2 | 5 |
| **Window 20** | 479.1-483.1 | 4 | 5 |
| **Window 21** | 481.8-485.8 | 4 | 5 |
| **Window 22** | 484.5-488.6 | 4.1 | 5 |
| **Window 23** | 487.6-491.6 | 4 | 5 |
| **Window 24** | 490.3-494.9 | 4.6 | 5 |
| **Window 25** | 493.9-499 | 5.1 | 5 |
| **Window 26** | 498-503.5 | 5.5 | 5 |
| **Window 27** | 502.5-507.5 | 5 | 5 |
| **Window 28** | 506.5-512 | 5.5 | 5 |
| **Window 29** | 511-516.1 | 5.1 | 5 |
| **Window 30** | 515.1-520.1 | 5 | 5 |
| **Window 31** | 519.1-523.7 | 4.6 | 5 |
| **Window 32** | 522.7-527.8 | 5.1 | 5 |
| **Window 33** | 526.8-530.9 | 4.1 | 5 |
| **Window 34** | 529.9-534.1 | 4.2 | 5 |
| **Window 35** | 533.1-537.1 | 4 | 5 |
| **Window 36** | 535.8-539.8 | 4 | 5 |
| **Window 37** | 538.5-542.5 | 4 | 5 |
| **Window 38** | 540.7-544.7 | 4 | 5 |
| **Window 39** | 543.7-547.7 | 4 | 5 |
| **Window 40** | 546.7-550.7 | 4 | 5 |
| **Window 41** | 549.7-553.7 | 4 | 5 |
| **Window 42** | 552.7-556.7 | 4 | 5 |
| **Window 43** | 555.7-559.7 | 4 | 5 |
| **Window 44** | 558.7-562.7 | 4 | 5 |
| **Window 45** | 561.7-565.7 | 4 | 5 |
| **Window 46** | 564.7-568.7 | 4 | 5 |
| **Window 47** | 567.7-571.7 | 4 | 5 |
| **Window 48** | 570.7-574.7 | 4 | 5 |
| **Window 49** | 573.7-577.7 | 4 | 5 |
| **Window 50** | 576.7-580.7 | 4 | 5 |
| **Window 51** | 579.7-583.7 | 4 | 5 |
| **Window 52** | 582.7-586.7 | 4 | 5 |
| **Window 53** | 585.7-589.7 | 4 | 5 |
| **Window 54** | 588.7-592.7 | 4 | 5 |
| **Window 55** | 591.7-595.7 | 4 | 5 |
| **Window 56** | 594.7-598.7 | 4 | 5 |
| **Window 57** | 597.7-601.7 | 4 | 5 |
| **Window 58** | 600.7-604.7 | 4 | 5 |
| **Window 59** | 603.7-607.7 | 4 | 5 |
| **Window 60** | 606.7-610.7 | 4 | 5 |
| **Window 61** | 609.7-613.7 | 4 | 5 |
| **Window 62** | 612.7-616.7 | 4 | 5 |
| **Window 63** | 615.7-619.7 | 4 | 5 |
| **Window 64** | 618.7-622.7 | 4 | 5 |
| **Window 65** | 620.9-624.9 | 4 | 5 |
| **Window 66** | 623.1-627.1 | 4 | 5 |
| **Window 67** | 625.8-629.8 | 4 | 5 |
| **Window 68** | 628.1-632.1 | 4 | 5 |
| **Window 69** | 630.8-634.8 | 4 | 5 |
| **Window 70** | 633-637 | 4 | 5 |
| **Window 71** | 635.7-639.7 | 4 | 5 |
| **Window 72** | 638.4-642.4 | 4 | 5 |
| **Window 73** | 641.1-645.1 | 4 | 5 |
| **Window 74** | 643.8-648 | 4.2 | 5 |
| **Window 75** | 647-651 | 4 | 5 |
| **Window 76** | 649.7-653.7 | 4 | 5 |
| **Window 77** | 652.4-656.5 | 4.1 | 5 |
| **Window 78** | 655.5-659.7 | 4.2 | 5 |
| **Window 79** | 658.7-663.3 | 4.6 | 5 |
| **Window 80** | 662.3-666.9 | 4.6 | 5 |
| **Window 81** | 665.9-670.5 | 4.6 | 5 |
| **Window 82** | 669.5-674.1 | 4.6 | 5 |
| **Window 83** | 673.1-677.7 | 4.6 | 5 |
| **Window 84** | 676.7-681.3 | 4.6 | 5 |
| **Window 85** | 680.3-684.9 | 4.6 | 5 |
| **Window 86** | 683.9-688.5 | 4.6 | 5 |
| **Window 87** | 687.5-692.1 | 4.6 | 5 |
| **Window 88** | 691.1-696.1 | 5 | 5 |
| **Window 89** | 695.1-700.6 | 5.5 | 5 |
| **Window 90** | 699.6-704.7 | 5.1 | 5 |
| **Window 91** | 703.7-708.7 | 5 | 5 |
| **Window 92** | 707.7-712.3 | 4.6 | 5 |
| **Window 93** | 711.3-715.5 | 4.2 | 5 |
| **Window 94** | 714.5-719.1 | 4.6 | 5 |
| **Window 95** | 718.1-722.7 | 4.6 | 5 |
| **Window 96** | 721.7-725.8 | 4.1 | 5 |
| **Window 97** | 724.8-729.4 | 4.6 | 5 |
| **Window 98** | 728.4-733 | 4.6 | 5 |
| **Window 99** | 732-736.2 | 4.2 | 5 |
| **Window 100** | 735.2-739.2 | 4 | 5 |
| **Window 101** | 737.9-742 | 4.1 | 5 |
| **Window 102** | 741-745 | 4 | 5 |
| **Window 103** | 743.7-747.9 | 4.2 | 5 |
| **Window 104** | 746.9-751 | 4.1 | 5 |
| **Window 105** | 750-754 | 4 | 5 |
| **Window 106** | 752.7-756.9 | 4.2 | 5 |
| **Window 107** | 755.9-760 | 4.1 | 5 |
| **Window 108** | 759-763.2 | 4.2 | 5 |
| **Window 109** | 762.2-766.2 | 4 | 5 |
| **Window 110** | 764.9-769 | 4.1 | 5 |
| **Window 111** | 768-772.6 | 4.6 | 5 |
| **Window 112** | 771.6-775.8 | 4.2 | 5 |
| **Window 113** | 774.8-779.4 | 4.6 | 5 |
| **Window 114** | 778.4-783 | 4.6 | 5 |
| **Window 115** | 782-786.1 | 4.1 | 5 |
| **Window 116** | 785.1-789.3 | 4.2 | 5 |
| **Window 117** | 788.3-792.4 | 4.1 | 5 |
| **Window 118** | 791.4-795.6 | 4.2 | 5 |
| **Window 119** | 794.6-799.2 | 4.6 | 5 |
| **Window 120** | 798.2-802.8 | 4.6 | 8 |
| **Window 121** | 801.8-807.3 | 5.5 | 8 |
| **Window 122** | 806.3-811.3 | 5 | 8 |
| **Window 123** | 810.3-815.8 | 5.5 | 8 |
| **Window 124** | 814.8-820.3 | 5.5 | 8 |
| **Window 125** | 819.3-824.8 | 5.5 | 8 |
| **Window 126** | 823.8-829.3 | 5.5 | 8 |
| **Window 127** | 828.3-833.8 | 5.5 | 8 |
| **Window 128** | 832.8-838.3 | 5.5 | 8 |
| **Window 129** | 837.3-843.3 | 6 | 8 |
| **Window 130** | 842.3-848.2 | 5.9 | 8 |
| **Window 131** | 847.2-853.2 | 6 | 8 |
| **Window 132** | 852.2-857.7 | 5.5 | 8 |
| **Window 133** | 856.7-861.7 | 5 | 8 |
| **Window 134** | 860.7-866.2 | 5.5 | 8 |
| **Window 135** | 865.2-870.7 | 5.5 | 8 |
| **Window 136** | 869.7-875.2 | 5.5 | 8 |
| **Window 137** | 874.2-880.2 | 6 | 8 |
| **Window 138** | 879.2-884.7 | 5.5 | 8 |
| **Window 139** | 883.7-889.2 | 5.5 | 8 |
| **Window 140** | 888.2-894.1 | 5.9 | 8 |
| **Window 141** | 893.1-898.6 | 5.5 | 8 |
| **Window 142** | 897.6-903.1 | 5.5 | 8 |
| **Window 143** | 902.1-908.1 | 6 | 8 |
| **Window 144** | 907.1-913 | 5.9 | 8 |
| **Window 145** | 912-919.3 | 7.3 | 8 |
| **Window 146** | 918.3-927.9 | 9.6 | 8 |
| **Window 147** | 926.9-936.4 | 9.5 | 8 |
| **Window 148** | 935.4-945.4 | 10 | 8 |
| **Window 149** | 944.4-955.3 | 10.9 | 8 |
| **Window 150** | 954.3-965.2 | 10.9 | 8 |
| **Window 151** | 964.2-975.6 | 11.4 | 8 |
| **Window 152** | 974.6-986.8 | 12.2 | 8 |
| **Window 153** | 985.8-999.4 | 13.6 | 8 |
| **Window 154** | 998.4-1011.6 | 13.2 | 10 |
| **Window 155** | 1010.6-1023.3 | 12.7 | 10 |
| **Window 156** | 1022.3-1036.8 | 14.5 | 10 |
| **Window 157** | 1035.8-1051.6 | 15.8 | 10 |
| **Window 158** | 1050.6-1067.4 | 16.8 | 10 |
| **Window 159** | 1066.4-1084.5 | 18.1 | 10 |
| **Window 160** | 1083.5-1103.4 | 19.9 | 10 |
| **Window 161** | 1102.4-1121.4 | 19 | 10 |
| **Window 162** | 1120.4-1139.8 | 19.4 | 10 |
| **Window 163** | 1138.8-1159.6 | 20.8 | 10 |
| **Window 164** | 1158.6-1181.7 | 23.1 | 10 |
| **Window 165** | 1180.7-1205.1 | 24.4 | 10 |
| **Window 166** | 1204.1-1228 | 23.9 | 10 |
| **Window 167** | 1227-1249.6 | 22.6 | 10 |
| **Window 168** | 1248.6-1252.6 | 4 | 10 |

**Reference:**

1. Gillet, L.C.; Navarro, P.; Tate, S.; Röst, H.; Selevsek, N.; Reiter, L.; Bonner, R.; Aebersold, R. Targeted data extraction of the MS/MS spectra generated by data-independent acquisition: a new concept for consistent and accurate proteome analysis. *Molecular & Cellular Proteomics* **2012**, *11*.

2. Tang, W.H.; Shilov, I.V.; Seymour, S.L. Nonlinear fitting method for determining local false discovery rates from decoy database searches. *Journal of proteome research* **2008**, *7*, 3661-3667, doi:10.1021/pr070492f.

3. Sennels, L.; Bukowski-Wills, J.C.; Rappsilber, J. Improved results in proteomics by use of local and peptide-class specific false discovery rates. *BMC bioinformatics* **2009**, *10*, 179, doi:10.1186/1471-2105-10-179.

4. Lambert, J.-P.; Ivosev, G.; Couzens, A.L.; Larsen, B.; Taipale, M.; Lin, Z.-Y.; Zhong, Q.; Lindquist, S.; Vidal, M.; Aebersold, R. Mapping differential interactomes by affinity purification coupled with data-independent mass spectrometry acquisition. *Nature methods* **2013**.

5. Collins, B.C.; Gillet, L.C.; Rosenberger, G.; Röst, H.L.; Vichalkovski, A.; Gstaiger, M.; Aebersold, R. Quantifying protein interaction dynamics by SWATH mass spectrometry: application to the 14-3-3 system. *Nature methods* **2013**.
