# Supplementary material for: Proteomic Analyses Reveal New Insights on the Antimicrobial Mechanisms of Chitosan Biopolymers and Their Nanosized Particles against Escherichia coli
Source: Int J Mol Sci. 2019 Dec 28;21(1):225. doi: 10.3390/ijms21010225 (PMC6981525; doi:10.3390/ijms21010225)
Supplement: Supplementary file 1 [file ijms-21-00225-s001.zip › Suplemmentary material_ Figure_ Subimitted 27-12.docx]

**Supplementary material**

**Supplementary Figure S1.** Distribution of the quantified protein by GO terms. Panel A, molecular function; panel B, pathway; panel C, protein classes; panel D, cellular components and panel E, biological process.

**Supplementary Figure S2**. Graphical representation of the STRING analysis, the set of six accumulated proteins, their identified protein-protein interaction using as a minimum required interaction score of 0.4 taking into account active interaction source parameters such as bibliographic citations, data co-expression, chromosomal neighborhood, genetic fusion and co-occurrence.

**Supplementary Figure S3.** Multivariate statistical analysis of biological processes globally affected by chitosan forms. PLS-DA test (PC=5) of all nano LC-MS/MS quantified proteins). Dots within each area represent the biological replicates of each condition (n=4). Discriminant proteins with VIP scores above 1. The colored boxes on the right indicate the relative amounts of the corresponding proteins in each assessed experimental condition.


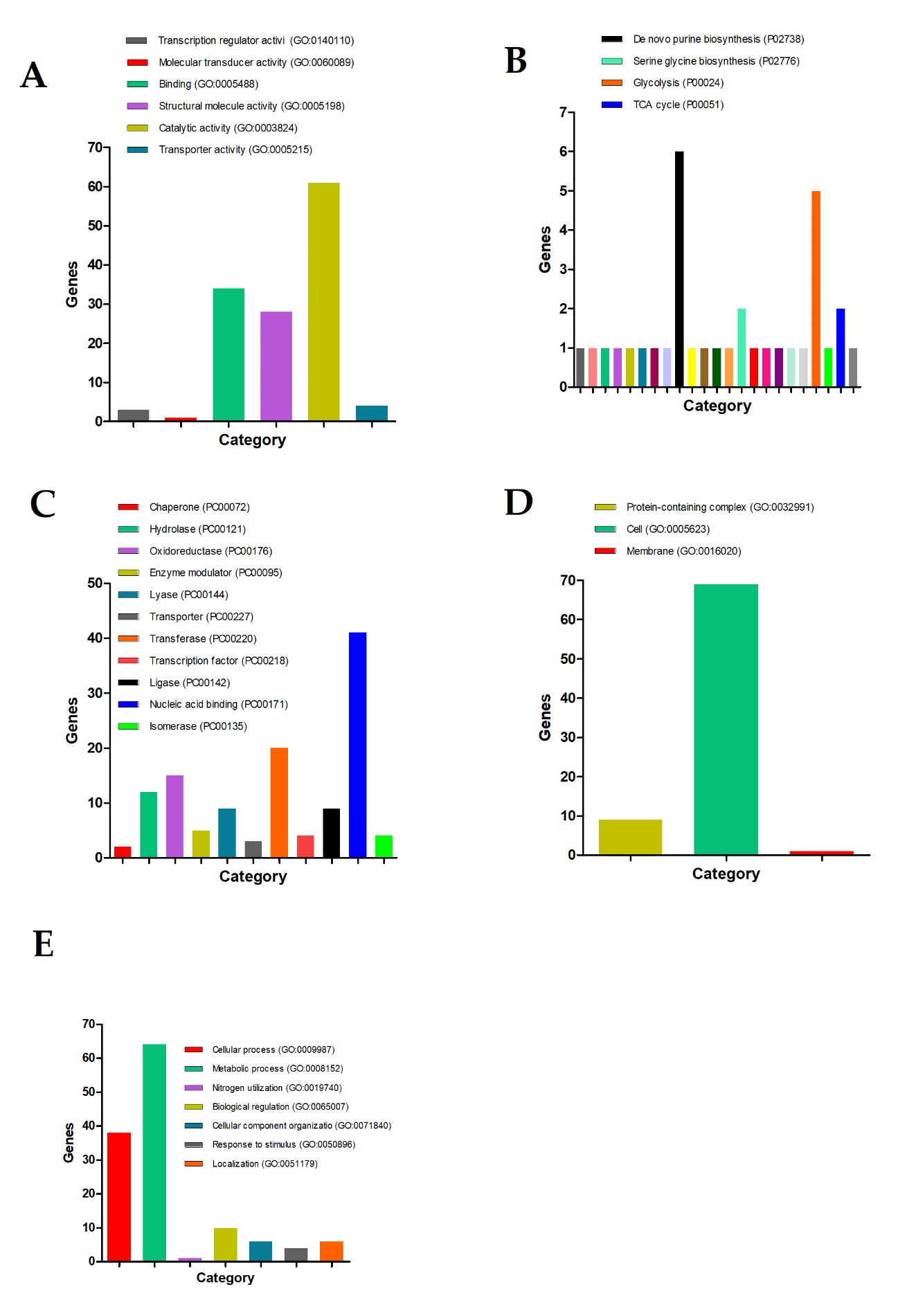


**Supplementary Figure S1.** Distribution of the quantified protein by GO terms. Panel A, molecular function; panel B, pathway; panel C, protein classes; panel D, cellular components and panel E, biological process.

**
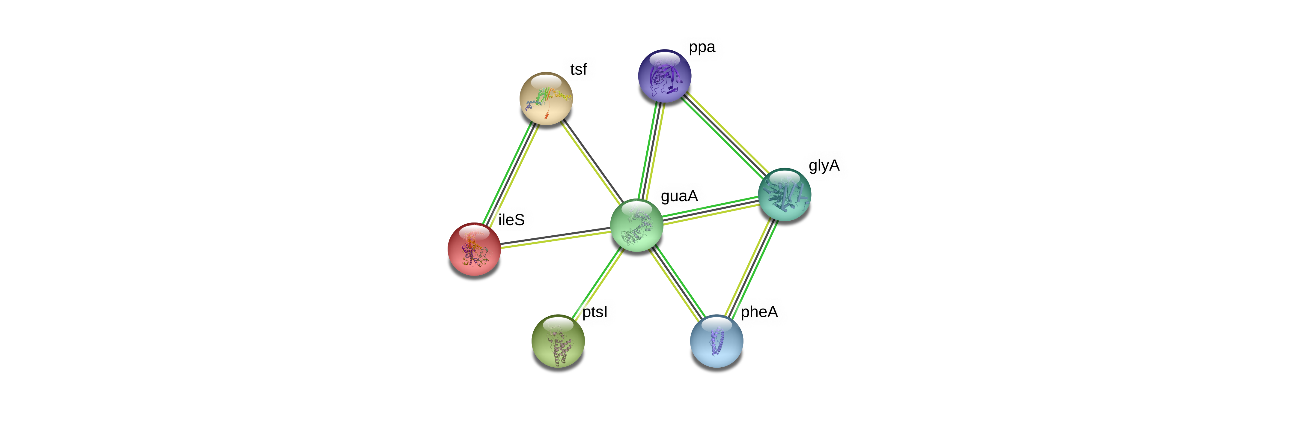
Supplementary Figure S2**. Graphical representation of the STRING analysis, the set of six accumulated proteins, their identified protein-protein interaction using as a minimum required interaction score of 0.4 taking into account active interaction source parameters such as bibliographic citations, data co-expression, chromosomal neighborhood, genetic fusion and co-occurrence.


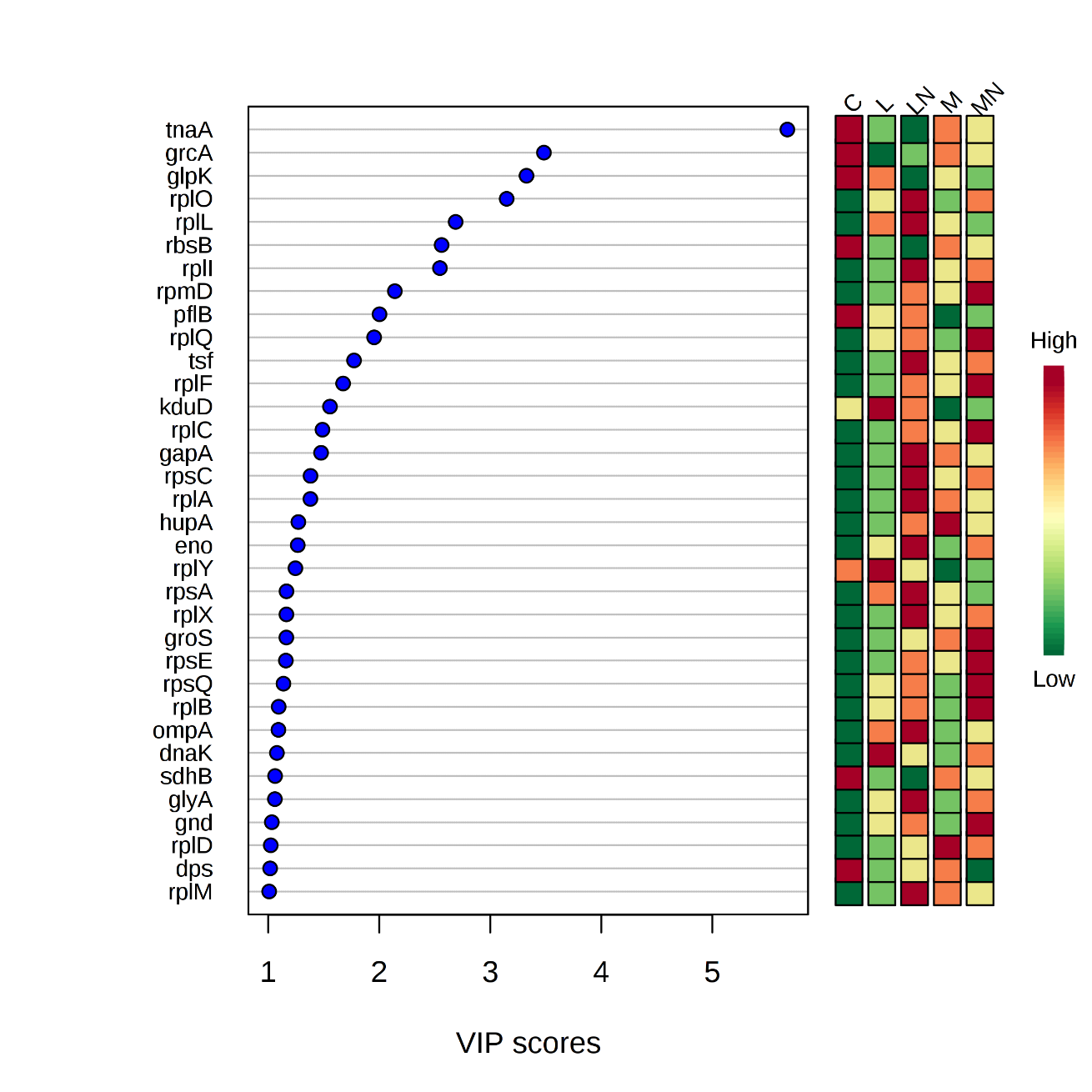


**Supplementary Figure S3.** Multivariate statistical analysis of biological processes globally affected by chitosan forms. PLS-DA test (PC=5) of all nano LC-MS/MS quantified proteins). Dots within each area represent the biological replicates of each condition (n=4). Discriminant proteins with VIP scores above 1. The colored boxes on the right indicate the relative amounts of the corresponding proteins in each assessed experimental condition.
